# Maternal Malaria and Gravidity Interact to Modify Infant Susceptibility to Malaria

**DOI:** 10.1371/journal.pmed.0020407

**Published:** 2005-11-08

**Authors:** Theonest K Mutabingwa, Melissa C Bolla, Jin-Long Li, Gonzalo J Domingo, Xiaohong Li, Michal Fried, Patrick E Duffy

**Affiliations:** 1MOMS Project, Malaria Antigen Discovery Program, Seattle Biomedical Research Institute, Seattle, Washington, United States of America,; **2**Gates Malaria Partnership, London School of Hygiene and Tropical Medicine, London, United Kingdom,; **3**National Institute for Medical Research, Muheza Designated District Hospital, Muheza-Tanga, Tanzania,; **4**Statistical Center for HIV/AIDS Research & Prevention, Department of Public Health Sciences, Fred Hutchinson Cancer Research Center, Seattle, Washington, United States of America,; **5**Department of Immunology, Walter Reed Army Institute of Research, Silver Spring, Maryland, United States of America; Centre for Geographic Medicine Research CoastKenya

## Abstract

**Background:**

In endemic areas, placental malaria due to *Plasmodium falciparum* is most frequent and severe in first-time mothers, and increases the risk of infant mortality in their offspring. Placental malaria may increase the susceptibility of infants to malaria parasitemia, but evidence for this effect is inconclusive.

**Methods and Findings:**

During 2002–2004, we monitored parasitemia in 453 infants, including 69 who were born to mothers with placental malaria, in a region of northeastern Tanzania where malaria transmission is intense. We used a Cox proportional hazards model to evaluate the time from birth to first parasitemia, and a generalized estimating equations logistic regression model to evaluate risk of any parasitemia throughout the first year of life. Compared with infants whose mothers did not have placental malaria at delivery (“PM-negative”), offspring of mothers with placental malaria at delivery (“PM-positive”) were 41% more likely to experience their first parasitemia at a younger age (adjusted hazard ratio [AHR] = 1.41, 95% confidence interval [CI] 1.01–1.99). The odds of parasitemia throughout infancy were strongly modified by the interaction between placental malaria and gravidity (*p* for interaction = 0.008, Type 3 likelihood ratio test). Offspring of PM-negative primigravidae had lower odds of parasitemia during infancy (adjusted odds ratio [AOR] = 0.67, 95% CI 0.50–0.91) than offspring of PM-negative multigravidae, and offspring of PM-positive primigravidae had the lowest odds (AOR = 0.21, 95% CI 0.09–0.47). In contrast, offspring of PM-positive multigravidae had significantly higher odds of parasitemia (AOR = 1.59, 95% CI 1.16–2.17).

**Conclusion:**

Although parasitemia is more frequent in primigravid than multigravid women, the converse is true in their offspring, especially in offspring of PM-positive women. While placental malaria is known to increase mortality risk for first-born infants, it surprisingly reduced their risk of parasitemia in this study. Placental malaria of multigravidae, on the other hand, is a strong risk factor for parasitemia during infancy, and therefore preventive antimalarial chemotherapy administered to multigravid women close to term may reduce the frequency of parasitemia in their offspring.

## Abstract 2

Children of mothers with placental malaria were more likely to exhibit parasitemia within their first year. First-born children were at a lower risk, regardless of whether their mothers had placental malaria.

## Introduction

The hallmark of pregnancy malaria due to *Plasmodium falciparum* is the accumulation of infected erythrocytes (IEs) in the placenta [1]. Placental IEs are a distinct parasite form that binds to chondroitin sulfate A (CSA) on syncytiotrophoblast and in intervillous spaces [2]. Placental IEs do not adhere to CD36, a ubiquitous receptor on the microvascular endothelium that commonly supports adhesion of IEs from non-pregnant individuals [2]. Adhesion to CSA allows parasites to sequester in the placenta, where dense accumulations can often occur with little or no parasitemia detectable in the peripheral blood [3].

Because CSA-binding parasites do not commonly infect non-pregnant individuals, women lack immunity to this parasite form prior to the first pregnancy [4]. In areas of stable malaria transmission, women acquire antibodies against placental parasites over successive pregnancies as a consequence of repeated exposures [4,5]. Maternal antibodies against placental IEs are associated with reduced risk of maternal parasitemia and improved pregnancy outcomes [6,7]. Because immunity is acquired over successive pregnancies, susceptibility to malaria is greatest during the first pregnancy and diminishes with increasing gravidity. Similarly, placental inflammation and the sequelae of pregnancy malaria, such as severe maternal anemia and low birth weight, are most frequent during first pregnancies [8–10].

Pregnancy malaria is estimated to cause tens of thousands or hundreds of thousands of infant deaths each year. However, these estimates are extrapolated from the incidence of malaria-related outcomes, such as low birth weight and maternal anemia that increase infant mortality risk [11,12], and the estimates sometimes differ from the real benefits observed in chemoprophylaxis trials. Low birth weight caused by pregnancy malaria has been estimated to cause 6% of infant deaths in sub-Saharan Africa [12], while antimalarial chemoprophylaxis delivered to pregnant women during the third trimester reduced infant mortality in The Gambia by 18% and 4% among offspring of primigravid and multigravid women, respectively [13]. In Malawi, chemoprophylaxis during pregnancy reduced infant mortality by 3%–5% [14]. The incomplete effectiveness of antimalarial regimens used in chemoprophylaxis studies [14] and the poor sensitivity of maternal peripheral blood slides or placental impression slides for detecting placental malaria (PM) [15] complicate studies that estimate the consequence of PM on infant outcomes.

Few studies have directly examined the effects of pregnancy malaria on infant health. A study conducted in southern Cameroon found no significant difference in the frequency of malaria between infants born to PM-positive mothers (46.5%) and infants born to PM-negative mothers (38.5%) during the first two years of life (χ^2^ = 0.24, *p* = 0.60) [16]. However, the age-specific prevalence of *P. falciparum* parasitemia was consistently higher between 4 and 18 mo of age among infants born to mothers with PM. The overall malaria-free survival rates were not significantly different between the two groups of infants, although a considerable decrease was observed between 5 and 8 mo of age among infants born to placenta-infected mothers [16]. In a subset analysis of the same infant cohort, cord serum reactivity against two CSA-binding parasite isolates (but not reactivity against other parasites) was related to a younger age at first parasitemia in the infant (*p* < 0.01) and to increased frequency of parasitemia during infancy [17]. The authors inferred that PM occurring during the last months of pregnancy may have increased antibody against CSA-binding parasites, and at the same time may have increased infant susceptibility to malaria due to immunologic tolerance or congenital transmission of parasites [17]. Results from these studies suggest that PM may increase malaria risk in the offspring, but further investigation is warranted. Therefore, we examined the effect of PM on the malaria susceptibility of offspring in a prospective cohort study of newborn infants in northeastern Tanzania.

## Methods

### Clinical and Laboratory Methods

The population for the present analysis was taken from the ongoing Mother-Offspring Malaria Study (MOMS) Project carried out in Muheza district, northeastern Tanzania, an area of intense malaria transmission. The entomologic inoculation rate in the area has been described as approximately 400 infective mosquito bites each year [18]. Recruitment of study participants began in September 2002. The study population for this report consisted of mother–infant pairs enrolled during delivery hospitalization at the Muheza Designated District Hospital. Pregnant women eligible for the study were between the ages of 18 and 45 y and had no evidence of chronic or debilitating illness, such as recent significant weight loss, chronic diarrhea, or history of chronic illness. Written informed consent was obtained from the mother prior to entering the study. Information on maternal characteristics (age, village of residence, gravidity) and history of intermittent presumptive therapy (IPT) during pregnancy was collected during in-person interviews by trained nurses at enrollment. Bed net usage was assessed by village health workers during clinical visits and onsite home visits.

Placental and infant parasitemia were defined as the presence of any parasites identified in a blood slide by microscopy. Placental blood samples were obtained after delivery by mechanically grinding full-thickness placental tissue [2]. Infant blood samples were collected by heel prick every 2 wk and at the time of any illness. Thick and thin smears were prepared for all samples; thin smears were fixed with methanol. Blood slides were stained for 10 min in 10% Giemsa, washed in tap water and air-dried, then examined using light microscopy at 100× magnification. Ten thousand red cells were examined in the thin smear before concluding that a placental blood slide was negative, and 100 high power fields were examined in the thick smear before concluding that a peripheral blood slide of an infant was negative.

Infants were seen at birth and at 2-wk intervals for full clinical examination and blood sampling, either at the Muheza Designated District Hospital or at the Project-sponsored mobile clinics that rotated through study villages on a weekly basis. Assistant medical officers performed the clinical examination and management of infants, under the supervision of a medical doctor. Infants with parasitemia and signs or symptoms suggestive of clinical malaria (most commonly fever ≥ 37.5 °C) were treated with 25 mg/kg amodiaquine over 3 d (10, 10, and 5 mg/kg on successive days). When amodiaquine failed to cure malaria, infants received quinine 10 mg/kg every 8 h for 7 d as “rescue” therapy. Infants were hospitalized and received quinine and other supportive care when malaria was complicated by severe anemia (Hgb < 5 g/dl), respiratory distress, seizures or coma, hyperparasitemia (> 2,500 parasites per 200 white blood cells), vomiting that precluded oral medications, or prostration.

Protocols for procedures used in this study were approved by the International Clinical Studies Review Committee of the Division of Microbiology and Infectious Diseases at the US National Institutes of Health, and ethical clearance was obtained from the Institutional Review Boards of Seattle Biomedical Research Institute and the National Institute for Medical Research in Tanzania.

### Analytical Population

Infants whose data are reported in this study were enrolled between September 2002 and September 2003, and were followed throughout the first year of life. During the recruitment period, 495 mothers gave consent to participate and delivered at the Muheza Designated District Hospital. Excluded from the analyses were ten twin infants, four stillbirths, three early neonatal deaths, and six neonates who were not examined beyond 4 wk of age because their families moved out of the study area. Infants with any evidence of HIV infection (mother seropositive on voluntary testing [*n* = 7], infant presented with suggestive signs or symptoms [*n* = 8], or suffered HIV/AIDS-related death [*n* = 4] during follow-up) were also excluded (*n* = 19 total). During the period of this study, HIV-seropositivity rates were 7.5% among women who agreed to undergo antenatal testing. After exclusions, a total of 453 infants remained for analyses presented in this report.

### Statistical Analysis

We examined the frequency distribution of maternal and infant characteristics according to PM status (parasitemia or no parasitemia) or gravidity (primigravid, secundigravid, or multigravid) using a chi-squared test for categorical variables and Student's *t*-test for continuous measurements. The Mann-Whitney test was used to assess differences in parasite density among women with PM. Median time to first parasitemia was obtained using Kaplan-Meier survival analysis and assessed with the log rank test. Follow-up time was calculated in weeks from birth until date of first positive diagnosis of parasitemia, date of death, date last known to be alive, September 30, 2004 (end of follow-up period), or 54 wk of age, whichever occurred first.

Cox regression analysis was used to determine whether the risk of first parasitemia was associated with PM at delivery. The dependent variable was time between birth and first positive diagnosis measured in weeks. The following variables were considered to be potential confounders a priori and were included in the model: gravidity (primigravid, secundigravid, multigravid), transmission season at time of birth (high or low), and bed net use in a household. According to the incidence of parasitemia among 3- to 12-mo-old infants in Muheza (unpublished data), the high transmission seasons in both 2003 and 2004 occurred from May through October, and the low transmission seasons occurred from November through April. Residence location was grouped into six categories by geographical area and distance to nearest health center. In order to allow for a different baseline hazard for each location, we used a stratified Cox regression model [19]. All variables were assessed for conformity to the proportional hazards assumption using the global p (PH) statistic based on the Schoenfeld residuals. The likelihood ratio test was used to assess for effect modification. Information on bed net use was missing for almost 14% of the study population, and the bed net data were therefore analyzed using a missing indicator, by assigning values of yes, no, and unknown.

To assess the effect of PM at delivery on the probability of infant parasitemia throughout infancy, generalized estimating equations logistic regression models were constructed with a first-order autoregressive working correlation structure (the analysis with exchangeable and m-dependent correlation structure produced nearly identical results for the data) and model-based standard errors [20]. This method allowed full use of the data while accounting for the correlation of repeated measurements over time. The multivariate model incorporated the main effect of PM at delivery, as well as gravidity and other confounding factors including bed net usage, age and transmission season at time of bloodsmear, location of residence, and transmission season at time of birth. A quadratic term for age ((age)^2^) was also included in the model because there appeared to be a curvilinear relationship between age and parasitemia. Offspring of secundigravidae versus multigravidae had similar probability of parasitemia during infancy (*p* > 0.98 and *p* > 0.38 when the mother was PM-positive or PM-negative, respectively), and therefore were combined for the final analysis. We assessed for effect modification between PM and gravidity by including an interaction term in the regression model and tested for significance using a Type 3 likelihood ratio test. Because the interaction term was significant (*p* = 0.008), results are presented stratified by primigravidae (no previous pregnancies) and secundi/multigravidae (one or more previous pregnancies). Treatment intensity (number of antimalarial treatments per week) was also assessed as a potential effect modifier in the different gravidity groups. Offspring of the four groups (PM-positive primigravid, PM-positive multigravid, PM-negative primigravid, PM-negative multigravid) did not significantly differ in the frequency of antimalarial treatments using the Kruskal-Wallis test (*p* = 0.20). Therefore, treatment intensity would not have a significant bias effect on the assessment of the relationship between gravidity, PM status, and other factors, and was not included in the final models.

All *p*-values are two-sided, and confidence intervals (CIs) were calculated at the 95% level. Statistical significance was set at *p* ≤ 0.05. Data analyses were conducted using S TATA version 8.0 (Stata Corporation, College Station, Texas, United States), Statview version 5.0, and SAS version 9.0 software (SAS Institute, Cary, North Carolina, United States).

## Results

PM was identified in 69 (15.2%) of the mothers. PM-positive women were more likely to be younger and to be experiencing first-time pregnancies than PM-negative women (Table 1). Among primigravid and secundigravid women, the frequency of PM was similar (24.2% and 23.6%, respectively), whereas only 5.6% of multigravid women had PM at delivery. The mean placental parasite density among PM-positive women was significantly higher for primigravidae (7.1% of red cells infected), compared with secundigravidae (2.2%) and multigravidae (1.6%) combined (Mann-Whitney, tied *p* = 0.03). Although the mean placental parasite density of PM-positive women did not differ significantly between birth months, the four highest density placental parasitemias occurred in three primigravidae (35.0%, 52.8%, and 76.5% parasitemias) and one secundigravida (23.3% parasitemia) during the high transmission seasons. Reported usage of sulfadoxine-pyrimethamine for IPT against malaria during pregnancy was high (69.7%), but did not significantly differ between PM-positive and PM-negative mothers. Reported IPT usage was also similar among different gravidity groups (63%, 73%, and 72% for primigravidae, secundigravidae, and multigravidae, respectively), as was bed net usage (62%, 59%, and 58%, respectively). Residence area was not associated with gravidity group (chi-squared test, *p* = 0.12).

**Table 1:**
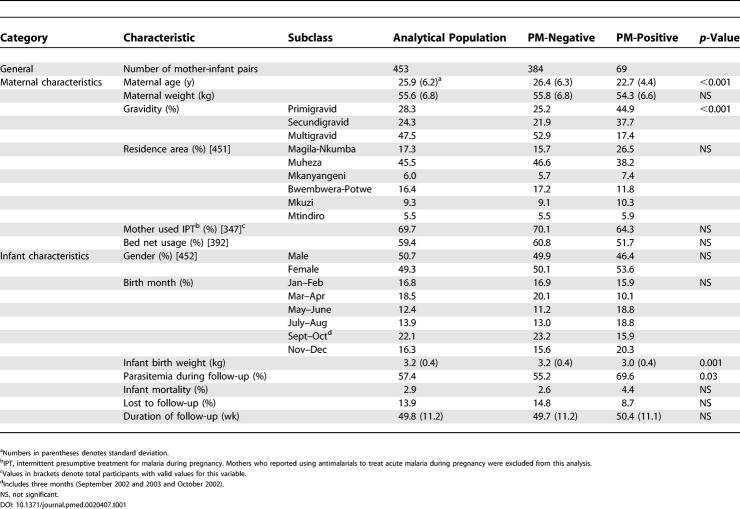
Characteristics of Study Participants

Offspring of PM-positive mothers were similar to those of PM-negative mothers by several measures, including gender, birth month, and residence (Table 1). Of the 445 infants whose birth weights were available, the mean birth weight was 3.2 kg (range 2.0–4.5 kg). Twenty-six (5.8%) weighed less than 2.5 kg, which is the standard criterion for low birth weight. Infants of PM-positive mothers weighed significantly less on average at birth than those of PM-negative mothers (Table 1). The overall mean duration of follow-up of infants was 50 wk (range 4–54) and did not differ by PM status at delivery (*p* = 0.67). Sixty-three (13.9%) infants were lost to follow-up between 4 and 54 wk of age (moved or parent[s] withdrew consent), including six of the 69 infants born to PM-positive mothers. Thirteen infants died before reaching 54 wk of age; three (4.4%) of those from PM-positive mothers and ten (2.6%) of those from PM-negative mothers.

### Risk of First Parasitemia

Two hundred and sixty (57.4%) infants in the study cohort experienced at least one episode of parasitemia by the age of 54 wk. Of the 193 infants who did not experience parasitemia, 138 (71.5%) were followed for at least 1 y. The median time to first parasitemia was 38 wk (95% CI 34–42) with a 75% probability of remaining free of parasitemia up to 18 wk of age. Of the 63 infants lost to follow-up, 17 (27.0%) presented with parasitemia before dropping out of the study.

The median time to first parasitemia for infants born to PM-positive mothers was 32 wk (95% CI 21–38) compared with 39 wk (95% CI 35–46) for offspring of PM-negative mothers (Figure 1). The effect of PM on the time to first parasitemia in the offspring appeared to vary across gravidity groups (Figure 2). For offspring of primigravidae, the median time to first parasitemia for infants born to PM-positive mothers was 34 wk (95% CI 23–52) compared with 38 wk (95% CI 30–49) for infants born to PM-negative mothers (*p* = 0.48). Among offspring of secundigravidae, the median time to first parasitemia for infants born to PM-positive mothers was 27 wk (95% CI 14–54) compared with 52 wk (95% CI 38–54) for infants born to PM-negative mothers (*p* = 0.06). Among offspring of multigravidae, the median time to first parasitemia for infants born to PM-positive mothers was 22 wk (95% CI 10–38) compared with 37 wk (95% CI 32–46) for infants born to PM-negative mothers (*p* = 0.01).

**Figure 1:**
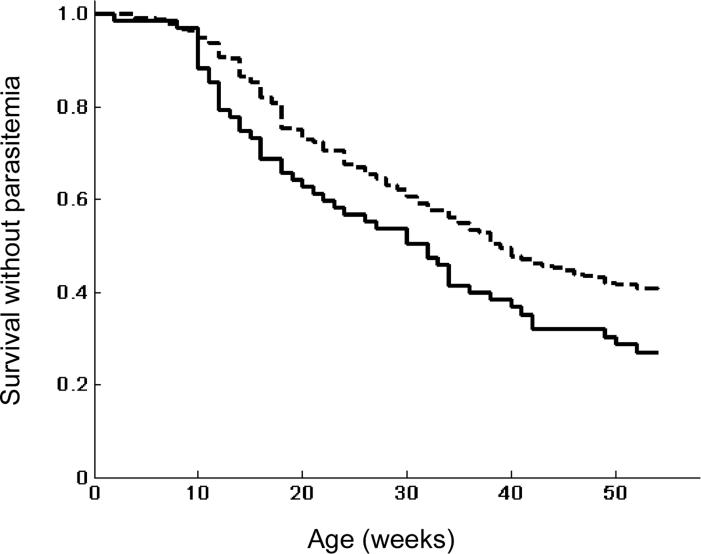
Kaplan-Meier Estimate of the Probability of Surviving without Parasitemia The graph shows the age at first parasitemia in offspring of PM-positive mothers (solid line) versus PM-negative mothers (dashed line). Infants born to PM-positive mothers experience their first parasitemia at a significantly younger age than infants of PM-negative mothers (log rank, *p* = 0.02).

**Figure 2:**
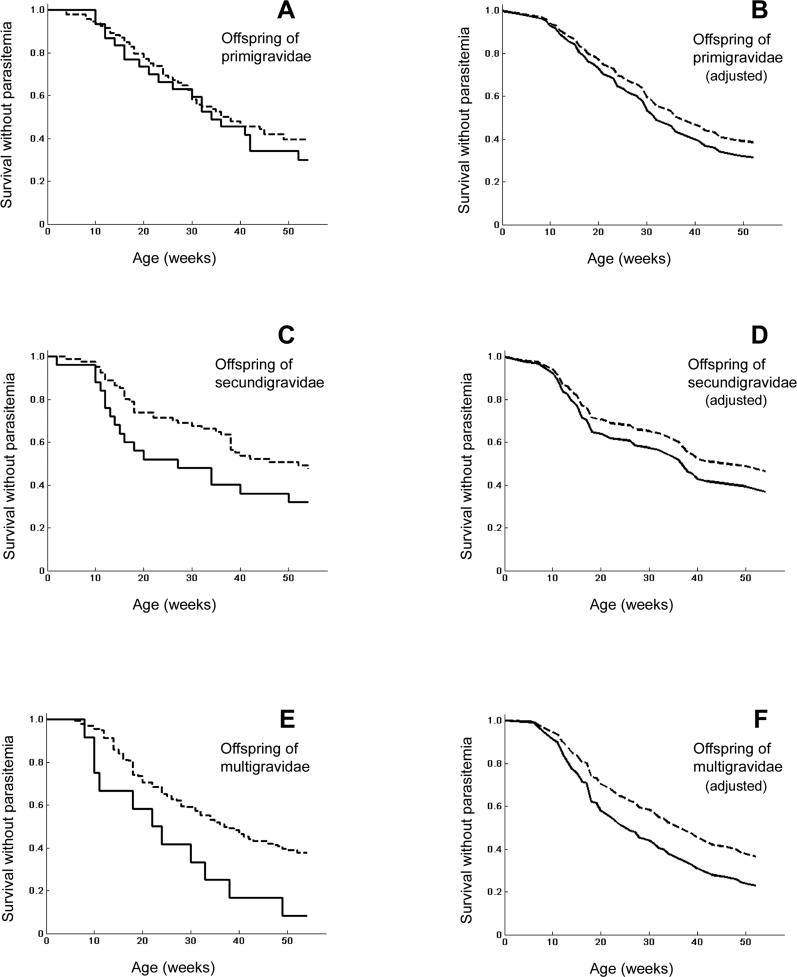
Kaplan-Meier Estimate of the Probability of Surviving without Parasitemia and Adjusted Estimate Using a Cox Model The graphs show the unadjusted (left panels) and adjusted (right panels) age at first parasitemia in first-born (A and B), second-born (C and D), or third-born or subsequent (E and F) offspring of PM-positive mothers (solid lines) versus PM-negative mothers (dashed lines). PM is associated with a significantly younger age at the time of first parasitemia among offspring of multigravidae (log rank, *p* = 0.01), but not among offspring of primigravidae (log rank, *p* = 0.48) or secundigravidae (log rank, *p* = 0.06). Adjustment estimates using a Cox model did not change statistical significance in any gravidity group.

Using stratified Cox regression, the estimated hazard ratio of first parasitemia for infants born to PM-positive mothers was 1.41 (95% CI 1.01–1.99) times that of infants born to PM-negative mothers, after adjustment for gravidity, transmission season at time of birth, area of residence, and bed net usage (Table 2). Although the test for interaction between gravidity and placental infection was not statistically significant (likelihood ratio test, *p* = 0.27), the effect of PM on infant risk appeared to vary across gravidity groups after adjustment for potential confounders (Figure 2B, 2D, and 2F). Among offspring of multigravid women, the risk of first parasitemia for infants born to PM-positive mothers was more than twice that of infants born to PM-negative mothers, and remained significant after adjustment for transmission season at time of birth, area of residence, and bed net usage (adjusted hazard ratio [AHR] = 2.20, 95% CI 1.15–4.20). The risk of first parasitemia among offspring of primigravid or secundigravid women was not significantly associated with PM after adjustment for potential confounding factors (AHR = 1.15, 95% CI 0.66–2.00, AHR = 1.15, 95% CI 0.61–2.17, respectively).

**Table 2:**
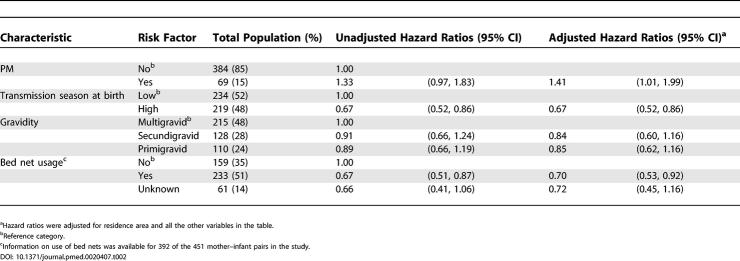
Unadjusted and Adjusted Hazard Ratios for Risk of First Parasitemia during Infancy

### Risk of Any Parasitemia

The age-specific prevalence of parasitemia during infancy differed by gravidity and PM status of the mother regardless of transmission season (Figure 3). Blood-slide positivity was observed more frequently among offspring of PM-positive multigravidae than among offspring of PM-positive primigravidae, in all age groups above 2 months of age. This relationship was observed in analyses of all bloodsmears collected from infants in either the low transmission (Figure 3C) or high transmission season (Figure 3D).

**Figure 3:**
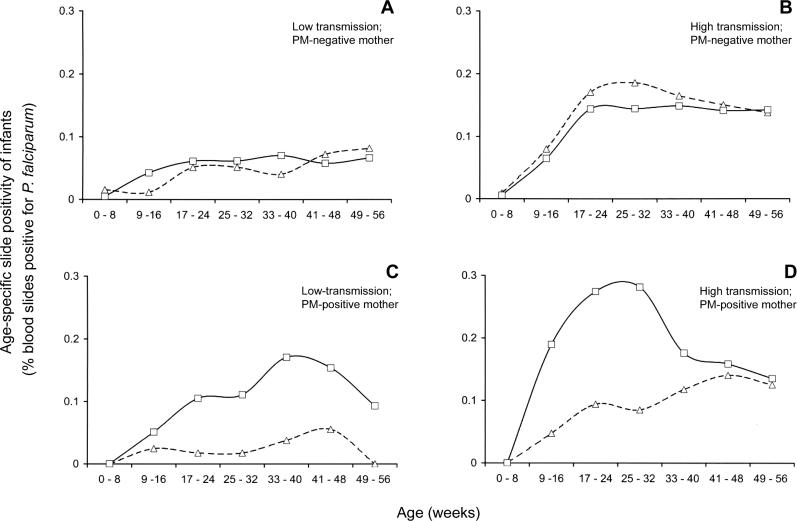
Age-Specific Parasite Positivity These graphs show age-specific parasite positivity of all blood slides obtained from infants born to primigravid women (dashed lines) versus secundigravid or multigravid women (solid lines). The frequency of parasitemia among offspring of PM-negative mothers (A and B) or offspring of PM-positive mothers (C and D) is presented according to whether the slides were collected during the low-transmission season (A and C) or high-transmission season (B and D). Low transmission of malaria around Muheza occurs from November through April, and high transmission occurs from May through October. Parasitemia was more frequent among the offspring of secundigravid and multigravid women versus primigravid women when PM was present at delivery. This relationship was observed in all age groups except neonates, and was observed in both low- (C) and high- (D) transmission seasons. Figures represent frequency of positivity for all slides collected during the study period, including slides collected subsequent to treatment.

Results from generalized estimating equations logistic regression analysis of the interaction between gravidity and PM on the odds of parasitemia are presented in Table 3. After adjustment for age, transmission season at birth and at time of bloodsmear, bednet usage, and residence, offspring of PM-negative primigravid women had significantly reduced odds of parasitemia during the first year of life (adjusted odds ratio [AOR] = 0.67, 95% CI 0.50–0.91), and the odds were lowest among offspring of PM-positive primigravid women (AOR = 0.21, 95% CI 0.09–0.47). In contrast, PM at delivery in multigravid women was associated with significantly increased odds of parasitemia in their offspring (AOR = 1.59, 95% CI 1.16–2.17).

**Table 3:**
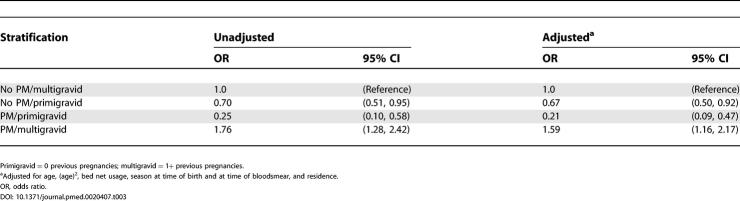
Unadjusted and Adjusted Odds of Any Parasitemia during Infancy Associated with Placental Malaria, Stratified by Gravidity

## Discussion

In this prospective cohort study of 453 mother–offspring pairs, both PM and maternal gravidity influenced the risk of *P. falciparum* parasitemia during infancy. Overall, offspring of PM-positive mothers experienced their first parasitemia at a significantly younger age than offspring of PM-negative mothers, and the risk of early first parasitemia was highest among offspring of PM-positive multigravidae. The risk of any parasitemia during infancy was strongly modified by an interaction between PM and gravidity. Offspring of PM-negative primigravidae had a decreased risk of parasitemia, while offspring of PM-positive primigravidae had the lowest risk. In contrast, offspring of PM-positive multigravidae had an increased risk of parasitemia.

Our results are the first to identify an effect of maternal gravidity to modify malaria risk in offspring during infancy. An earlier study in southern Cameroon observed an increased age-specific prevalence of *P. falciparum* in infants born to PM-positive mothers [16], but did not assess the effect of gravidity. Surprisingly, in the present study, PM decreased malaria risk in offspring of primigravidae but increased risk in offspring of other gravid groups. The opposing effects of PM in different gravid groups observed in the present study may explain why earlier studies failed to find significant relationships between PM and malaria risk during the first two years of life [16].

Because PM had been suggested to increase susceptibility during infancy [16,21], we expected offspring of PM-positive primigravidae to be at highest risk, since PM is most frequent and placental parasite densities are highest during primigravid pregnancies. In Malawi, high placental parasite densities were associated with increased risk of cord blood parasitemia [22], supporting the conjecture that congenital malaria could account for the relationship between PM and susceptibility of infants [17]. Although we expected PM to increase susceptibility in offspring of primigravid women to the greatest degree, we observed the opposite pattern. Our data are consistent with those of an earlier study [17] showing that cord blood levels of antibody against CSA-binding parasites were positively related to child susceptibility to malaria. Antibody against CSA-binding parasites is highest in multigravid women [4], and we find that offspring of multigravid women have the highest risk of parasitemia. The results from the earlier study were interpreted to suggest that antibody against CSA-binding parasites reflected a recent maternal parasitemia that may induce immunologic tolerance or congenital malaria [17], or else reflected an increased exposure to malaria for both the mother and the offspring [23]. However, neither interpretation would fully explain our finding that an interaction between gravidity and PM modifies infant susceptibility to parasitemia.

PM during the antenatal period may be influencing infant outcomes, separate from any effect of neonatal exposure occurring at delivery, and could explain the reduced risk of parasitemia among offspring of PM-negative primigravidae. In areas of stable transmission, primigravid women are parasitemic more frequently [24], and experience higher parasite densities, more severe inflammatory responses [8], and greater clinical sequelae [13,25] than women in other gravid groups. In the current cohort, 24.2% of primigravid women had PM with a mean parasite density of 7.1% at the time of delivery, compared with 5.6% of multigravid women with a mean parasite density of 1.6%, reflecting the susceptibility of primigravid women. Although we cannot exclude the possibility that an intrinsic effect of gravidity may modify malaria susceptibility of offspring, we favor the explanation that PM during the antenatal period is modulating the susceptibility of the offspring, because PM at delivery is having a similar (but more profound) effect. We propose that differences in the load of malarial antigen or the intensity of the inflammatory response may account for the differences observed between gravid groups, but this remains to be determined.

Factors such as area of residence, season of observation, and HIV may increase the risk of parasitemia in both mother and offspring, and therefore may confound an analysis of maternal parasitemia and infant susceptibility. However, our adjusted analyses suggest that these confounding factors would not fully explain the results. Although the incidence of parasitemia during infancy differed significantly between villages in this study (unpublished data), the frequency of PM did not (Table 1). Nor did the frequency of PM vary significantly by birth month (Table 1). While infants born during the low transmission season had a higher risk of parasitemia (presumably due to the seasonality of transmission), the increased frequency of parasitemia observed in offspring of PM-positive secundi- and multigravidae was apparent in both high and low transmission seasons (Figure 3), suggesting that the relationship between PM and infant susceptibility in the different gravidity groups was independent of seasonality. The intensive longterm follow-up of infants was likely to identify the majority of infants with HIV infection, as progression to disease is rapid in infants, especially African infants [26]. Eleven of the 12 offspring of PM-positive multigravidae included in our analyses had been followed for 18 mo or more, and none of the 12 experienced signs or symptoms of HIV infection. In sum, confounding factors associated with risk of parasitemia do not fully explain the gravidity-dependent relationships observed in this study, and specifically run counter to the observation that offspring of PM-positive primigravidae have a decreased risk of parasitemia.

Behavioral and environmental factors may vary with gravidity and could account for some gravidity-dependent relationships. An increased number of children in multigravid households would possibly attract more mosquitoes or increase the proportion of mosquitoes that are infective, thereby increasing malaria exposure among offspring of multigravidae. However, this would not explain why PM at delivery decreases malaria risk in offspring of primigravidae. Conversely, the increased frequency of fever in primigravid women may cause them to consume more antimalarials, which could be transferred to the fetus or infant and reduce infant parasitemia. However, reported usage of antimalarials during pregnancy was similar among the different gravidity groups. Furthermore, PM was associated with a risk of parasitemia throughout infancy, and is unlikely to be accounted for by ingestion of antimalarials at term.

Our findings suggest that PM decreases susceptibility in the offspring of primigravidae, and increases susceptibility in the offspring of multigravidae. Among primigravidae, the pronounced inflammatory response to PM could confer protection, for example by reducing parasitemia [27] and thereby preventing congenital transmission. Alternatively, sensitization of the fetal or neonatal immune system may be modulating immunity in a way that modifies susceptibility to parasitemia, such as by inducing regulatory T (Treg) cells, which have been related to malaria susceptibility in rodents [28,29]. Inflammatory signals inhibit the development of Treg cells [30], and thus the inflammatory responses that are common in primigravid but not multigravid women with PM could lead to differential effects of sensitization in their offspring. Separately, studies in Kenya have shown that antibodies against the *P. falciparum* MSP-1 antigen were lower among offspring of PM-positive mothers [21], and it will be valuable to determine whether anti-MSP-1 antibody responses in infants vary according to both PM and maternal gravidity. Future studies should seek to better understand congenital transmission of *P. falciparum,* as well as the effect of placental inflammation to modulate the infant's antibody and cellular responses against the parasite.

The findings of the present study establish a conundrum. PM at delivery greatly reduced the odds of parasitemia in offspring born to primigravid women, yet earlier studies reported that PM was the strongest risk factor for severe morbidity [31–34] and mortality of first-born infants [13]. More research is needed to understand the impact of PM on malaria-related and other clinical outcomes during infancy. The gravidity-dependent pattern of infant susceptibility identified here may provide a useful tool to study the relationship between the frequency of *P. falciparum* parasitemia and disease in young children.

In summary, PM decreases the risk of parasitemia among offspring of primigravidae but increases the risk among offspring of multigravidae. Traditionally, pregnancy malaria has been thought to affect the offspring primarily through low birth weight or maternal anemia, which are risk factors for neonatal and infant mortality. The present findings indicate that pregnancy malaria may also have prolonged effects on immunity and susceptibility to malaria in the offspring, and that these effects are strongly influenced by maternal gravidity. Further investigation of these relationships with assessment of other potential confounders, as well as their impact on infant morbidity and mortality, is needed with larger cohort studies. A preventive dose of antimalarial chemotherapy delivered to secundigravid or multigravid women in the last 1–2 wk before term may reduce *P. falciparum* parasitemia in their offspring, but more research is needed to predict its effect on malaria-related disease and death. The benefits of malaria prevention during pregnancy may extend beyond its effect on birth outcomes, and should be studied in randomized clinical trials with careful attention to PM status at delivery and outcomes during early childhood.

**Patient Summary**

**Background**

In areas where malaria is common, most adult women have acquired some level of immunity against the malaria parasite. However, during pregnancy, many experience a new type of malaria infection in their placenta. This placental malaria is particularly severe during first-time pregnancy. Pregnant women with placental malaria often give birth to babies with a low birth weight, and these babies have an increased risk of dying as infants.

**Why Was This Study Done?**

The connection between placental malaria and low birth weight is well established. However, it is not clear whether placental malaria makes it more likely for infants to get infected with malaria parasites themselves, and this is the question the researchers addressed in this study.

**What Did the Researchers Do and Find?**

They studied a total of 453 infants, of which 69 were born to mothers with placental malaria. Of those 69 infants, 31 were born to first-time mothers, and 38 to mothers who had given birth before. The researchers then followed those infants for a year and checked how often during that time they had parasites in their blood. Surprisingly, the researchers found that children followed one of two opposing patterns, depending on their birth order. Children from first-time mothers were less likely to have parasites in their blood, especially children whose mothers had placental malaria. Children from other mothers were more likely to have parasites in their blood, especially children whose mothers had placental malaria.

**What Does This Mean?**

These results suggest that both the number of previous pregnancies and placental malaria affect an infant's chances of developing malaria. It might be that in first-time mothers placental malaria stimulates the infant's immune system and this somehow protects the baby against malaria, but at present this is just speculation. Also, the study is relatively small, and the connections between the number of previous pregnancies, placental malaria, and the chances of an infant getting malaria need to be tested in other studies.

**Where Can I Find More Information Online?**

The following Web sites provide information about malaria.

Wikipedia pages on malaria:

http://en.wikipedia.org/wiki/Malaria

WHO pages on malaria, which contain a section on malaria and pregnancy:

http://www.who.int/topics/malaria/en/

MedlinePlus pages on malaria:

http://www.nlm.nih.gov/medlineplus/malaria.html

UNICEF page on malaria with links:

http://www.childinfo.org/eddb/Malaria/

## Abbreviations

AHR: adjusted hazard ratio
AOR: adjusted odds ratio
CI: confidence interval
CSA: chondroitin sulfate A
IE: infected erythrocyte
IPT: intermittent presumptive therapy
MOMS: Mother-Offspring Malaria Study
PM: placental malaria
